# DICER-LIKE2 Plays a Crucial Role in Rice Stripe Virus Coat Protein-Mediated Virus Resistance in Arabidopsis

**DOI:** 10.3390/v15112239

**Published:** 2023-11-10

**Authors:** Li Chen, Yanan Liu, Shuo Li, Yinghua Ji, Feng Sun, Baohong Zou

**Affiliations:** 1 The State Key Laboratory of Crop Genetics & Germplasm Enhancement and Utilization, Nanjing Agricultural University, Nanjing 210095, China; chenli@stu.njau.edu.cn; 2 Jiangsu Key Laboratory for Food Quality and Safety-State Key Laboratory Cultivation Base, Institute of Plant Protection, Jiangsu Academy of Agricultural Sciences, Nanjing 210014, China; yananliuw@163.com (Y.L.); lishuo@jaas.ac.cn (S.L.); jiyinghua@jaas.ac.cn (Y.J.)

**Keywords:** DCL2, DCL4, rice stripe virus, CP-mediated resistance, RNA silencing, small RNA deep sequence

## Abstract

Virus coat protein (CP)-mediated resistance is considered an effective antiviral defense strategy that has been used to develop robust resistance to viral infection. Rice stripe virus (RSV) causes significant losses in rice production in eastern Asia. We previously showed that the overexpression of RSV CP in Arabidopsis plants results in immunity to RSV infection, using the RSV-Arabidopsis pathosystem, and this CP-mediated viral resistance depends on the function of DCLs and is mostly involved in RNA silencing. However, the special role of DCLs in producing t-siRNAs in CP transgenic Arabidopsis plants is not fully understood. In this study, we show that RSV CP transgenic Arabidopsis plants with the *dcl2* mutant background exhibited similar virus susceptibility to non-transgenic plants and were accompanied by the absence of transgene-derived small interfering RNAs (t-siRNAs) from the CP region. The *dcl2* mutation eliminated the accumulation of CP-derived t-siRNAs, including those generated by other DCL enzymes. In contrast, we also developed RSV CP transgenic Arabidopsis plants with the *dcl4* mutant background, and these CP transgenic plants showed immunity to virus infection and accumulated comparable amounts of CP-derived t-siRNAs to CP transgenic Arabidopsis plants with the wild-type background except for a significant increase in the abundance of 22 nt t-siRNA reads. Overall, our data indicate that DCL2 plays an essential, as opposed to redundant, role in CP-derived t-siRNA production and induces virus resistance in RSV CP transgenic Arabidopsis plants.

## 1. Introduction

Plants have evolved different strategies to defend against viral invasion. Among these strategies, RNA silencing or RNA interference (RNAi) is considered the fundamental antiviral defense mechanism that has been used to increase resistance to viral infections [1]. In this process, virus-derived double-stranded (ds)RNA is generated as a viral genome replicative intermediate or by host RNA-dependent RNA polymerase (RDR) acting on aberrant viral single-strand RNA [2,3]. Subsequently, Dicer-like ribonucleases (DCLs) in plants recognize and convert these dsRNAs into 21–24-nucleotide viral small interfering RNAs (vsiRNAs) [4,5,6]. Eventually, one strand of the visRNAs is incorporated into Argonaute (AGO) proteins to form RNA-induced silencing complexes (RISCs). Antiviral RISCs are able to cleave or translationally suppress the targeted viral RNA through complementarity between the vsiRNA and the viral RNA [7,8]. In Arabidopsis thaliana, there are four types of DCLs that have all been associated with virus RNA silencing [5,6]. DCL1 primarily processes hairpin RNA into the 21 nt microRNA (miRNA) that is involved in plant growth, development and stress response [3]. Among vsiRNAs, the 21 nt vsiRNAs produced by DCL4 are the most dominant species and are involved in the canonical antiviral defense mechanism [5,9,10]. DCL2 generates 22 nt vsiRNAs that repress target viral RNA at the translational level and trigger the amplification of silencing signals more efficiently than 21 nt vsiRNAs [1,11,12]. DCL3 produces 24 nt vsiRNAs that direct transcriptional gene silencing by means of RNA-directed DNA methylation (RdDM) to suppress the transcription of DNA viruses [13,14]. In Arabidopsis plants, AGO1, AGO2, AGO4, AGO7, and AGO10 are associated with antiviral defense against various viruses by sorting vsiRNAs based on their 5′ terminal nucleotide [15,16,17,18].

Rice stripe virus (RSV) is the type species of the genus *Tenuivirus* and causes significant losses in rice production in eastern Asia [19,20,21]. In nature, RSV is transmitted from plant to plant by the small brown planthopper (SBPH) (*Laodelphax striatellus* Fallén) in a circulative and propagative manner [19,22]. Under laboratory conditions, RSV can be transmitted to Arabidopsis plants by means of viruliferous SBPH inoculation and causes severe disease symptoms, such as yellow stripes on leaves, severe stunting and even death [23,24]. The RSV genome contains four single-stranded negative-sense RNA segments, named RNA1, RNA2, RNA3 and RNA4, which encode seven open-reading frames (ORFs) using a negative or ambisense coding strategy [25]. RNA1 encodes an RNA-dependent RNA polymerase (RdRP) protein in the viral-complementary sense [26]. The other three segments are all bicistronic and ambisense, and each encodes two ORFs on the viral RNA and viral complementary strand (vcRNA). RNA2 encodes a weak RNA silencing suppressor NS2 [27] and a putative membrane glycoprotein Nsvc2 [28]. RNA3 encodes an RNA silencing suppressor NS3 [29] and a coat protein (CP) from vcRNA [30]. A disease-specific protein (SP) and a movement protein (NSvc4) are encoded by RNA4 from viral RNA and vcRNA, respectively [20,31]. To control the rice stripe disease caused by RSV, RNA silencing-associated resistance in transgenic plants has been shown to be a promising antiviral strategy. Transgenic rice plants expressing RNA silencing constructs targeting the SP, and CP genes of RSV conferred strong resistance to viral infection [32]. The overexpression of RSV CP or NSvc4 in transgenic rice plants provided resistance against the virus [33,34].

In a previous report, using the RSV-Arabidopsis pathosystem, we demonstrated that the overexpression of translatable and non-translatable RSV CP in Arabidopsis plants provided immunity to virus infection. Moreover, RSV CP-mediated virus resistance was associated with transgene-derived siRNAs (t-siRNAs) and depended on the function of endogenous DCLs [35]. However, which DCL genes are required for t-siRNA biosynthesis and virus resistance in CP transgenic Arabidopsis plants remains unknown. In the present study, we further investigated the roles of Arabidopsis DCL2 and DCL4 in RSV CP-mediated virus resistance. We developed RSV CP transgenic Arabidopsis plants with a *dcl2* or *dcl4* mutant background and found that DCL2 but not DLC4 was required for the accumulation of t-siRNAs and CP-mediated virus resistance. With the *dcl2* mutant background, the RSV CP transgenic plants showed the same susceptibility to virus infection as non-transgenic plants and a complete absence of accumulation of t-siRNA from CP loci. However, RSV CP transgenic plants in *dcl4* mutants exhibited immunity to RSV infection and accumulated high levels of transgene-siRNA (t-siRNA) (2.78% of total small RNA) with dominant sizes of 21 nt (37.92%) and 22 nt (49.68%). Collectively, our results demonstrate that Arabidopsis DCL2 plays a critical role in RSV CP-mediated antiviral defense by regulating the biogenesis of t-siRNAs.

## 2. Materials and Methods

### 2.1. Sources of Virus, Vectors, and Plant Materials

RSV-infected rice plants were collected from Jiangsu Province in China in 2019. Non-viruliferous planthoppers (*Laodelphax striatellus*) reared on *Oryza sativa* (Wuyujing No. 3) plants were collected and allowed an acquisition access period of at least 7 d on RSV-infected rice plants, following which planthoppers were transferred to rice plants. The viruliferous planthoppers were confirmed via RT-PCR assay [23].

*Arabidopsis thaliana* (Col-0) and Col-0;FMCP transgene seeds were described previously [35]. Seeds of *Arabidopsis thaliana* mutants *dcl2-1* (SALK_064627) and *dcl4-2e* (CS6954) were provided by ABRC. Arabidopsis plants were grown in potting soil in a growth chamber at 23 °C under 200 µmol m^−2^ s^−1^ illumination and 16 h light/8 h dark cycle conditions.

### 2.2. RSV Inoculation Assay

Viral inoculations were performed as described previously [35]. The Arabidopsis plants were infected at the 2-week stage with viruliferous planthoppers. After 4 days, the planthoppers were sprayed with insecticide, and the inoculated plants were placed in the growth chamber at 23 °C.

### 2.3. DNA Constructs and Transgenic Plants

The Arabidopsis transgene vector pBA-FM-CP was generated as described previously [35]. Arabidopsis *dcl2-1* and *dcl4-2e* plants were transformed with Agrobacterium tumefaciens ABI carrying pBA-FM-CP to generate RSV CP transformants using a floral dip method as described previously [36,37]. In the T1 and T2 generations, CP transgenic Arabidopsis plants were selected on standard MS medium containing 10 mg/L glufosinate ammonium (Sigma-Aldrich, St. Louis, MO, USA).

### 2.4. PCR Genotyping for Mutants

For homozygous *dcl2-1* genotyping, the T-DNA primers LB1.3, *dcl2-1-*LP and *dcl2-1-*RP, were designed on the website T-DNA Primer Design (http://signal-genet.salk.edu/tdnaprimers.2.html, accessed on 7 October 2022). Then, a PCR with these primers amplified genomic DNA from homozygous *dcl2-1* and wild-type Arabidopsis plants. Genotyping for homozygous *dcl4-2e* was performed as described previously [38], and PCR products were then sequenced using the sanger method.

### 2.5. Western Blot Assay

Plant total protein extraction and western blot analysis were performed as described previously [35]. Briefly, proteins were separated by 12% SDS-PAGE and transferred to PVDF membranes. The membranes were probed with primary anti-RSV SP (provided by Jianxiang Wu, Zhejiang University, Hangzhou, China), anti-Rubisco-L (Sangon Biotech, Shanghai, China), and anti-myc (Sigma-Aldrich, St. Louis, MO, USA), which was followed by the corresponding secondary antibodies conjugated to horseradish peroxidase (Beyotime Biotechnology, Shanghai, China). The blotted signal was visualized using ECL buffer (Vazyme, Nanjing, China) and recorded with a Tanon 5200 Luminescent Imaging Workstation according to the manufacturer’s manual (Tanon, Shanghai, China).

### 2.6. Quantitative Reverse-Transcription PCR (qRT-PCR)

Total RNA was extracted from Arabidopsis leaves with RNAiso Plus reagent (Takara, Dalian, China). For reverse transcription, 1 μg of RNA was used to synthesize cDNA using an iScriptTM cDNA Synthesis Kit (Bio-Rad, Hercules, CA, USA). qRT-PCR was then performed on a Bio-Rad iQ5 Real-Time PCR system (Bio-Rad, Hercules, CA, USA) with SsoFast EvaGreen Supermix (Bio-Rad, Hercules, CA, USA) with gene-specific primers (Supplementary Table S1). *EF1α* was used as an internal control, and each experiment included three independent biological replicates and three technical replicates.

### 2.7. Small RNA Deep Sequencing and Analysis

Total RNA samples were extracted from Col-0;FMCP, *dcl2-1*::FMCP and *dcl4-2e*::FMCP leaves using RNAiso Plus reagent (Takara, Dalian, China). Single-end mRNA libraries were generated using a Small RNA Sample Pre Kit (Illumina, San Diego, CA, USA) according to the manufacturer’s recommendations and were sequenced on an Illumina HiSeq SE50 platform at Novogene Corporation Inc. (Novogene, Tianjing, China). The quality of clean reads was checked using the FastQC program (v0.11.3). Then, clean data were mapped to the RSV CP (NCBI accession numbers: NC_003776.1) using Bowtie (v1.1.2), allowing up to one mismatch. Reads showing matches with the RSV CP sequences were retained and further analyzed using the CLC Genomics Workbench program (QIAGEN, Hilden, Germany), Perl scripts and Excel tools. The total small RNA reads mentioned in this study were the redundant reads. Small RNA-seq data were deposited in the National Genomics Data Center (part of the China National Center for Bioinformation) BioProject database with accession code PRJCA020309.

## 3. Results

### 3.1. Generation of RSV CP Transgenic Arabidopsis Plants with the dcl2 or dcl4 Mutant Background

**Figure 1:**
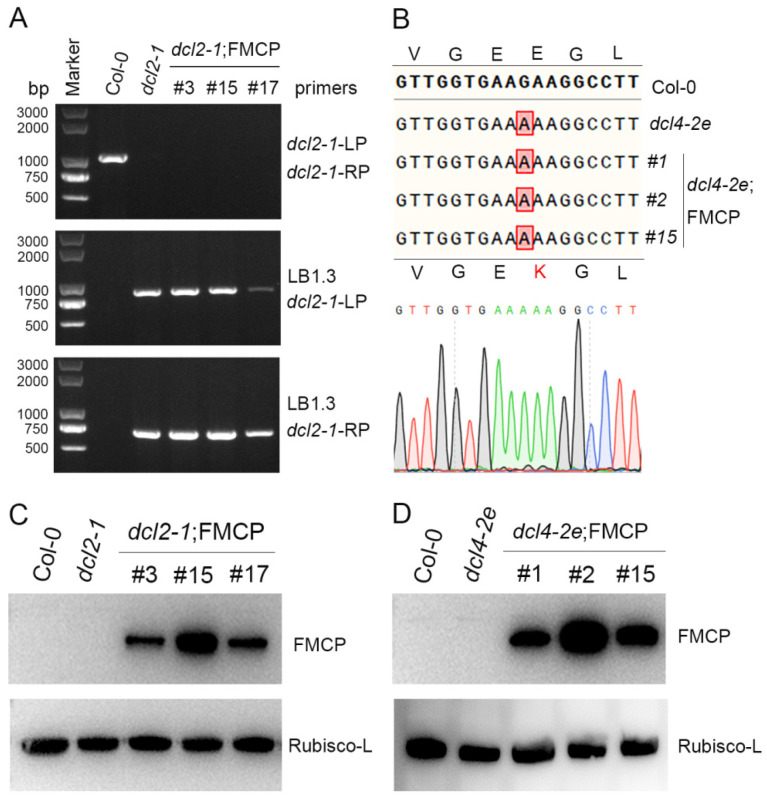
Molecular characterization of RSV CP transgenic Arabidopsis plants with a *dcl2* or *dcl4* mutant background. (**A**) Genotyping of *dcl2-1* T-DNA insertion mutant Arabidopsis plants. (**B**) DNA sequence analysis of *dcl4* mutated alleles identified from cloned PCR fragments of *dcl4-2e* mutant plants. (**C**) Western blot analysis of RSV CP protein accumulation in CP transgenic Arabidopsis plants with the *dcl2* mutant background using a myc-specific antibody. The Rubisco-L protein level served as a loading control. (**D**) Western blot analysis of RSV CP protein accumulation in CP transgenic Arabidopsis plants with the *dcl4* mutant background using a myc-specific antibody. The Rubisco-L protein level served as a loading control.

Our previous study indicated that RSV CP-mediated viral immunity in Arabidopsis plants depended on the function of endogenous DCLs [35]. To identify which DCL genes are required for RSV CP-mediated virus resistance, we generated RSV CP transgenic plants with the *dcl2-1* or *dcl4-2e* background using the pBA-FM-CP vector as described previously [35]. The *dcl2-1* mutant lines were null mutants generated by means of T-DNA insertion in *DCL2* (At3g03300) [39], and our genotyping PCR and sequence analysis results also confirmed that the *dcl2-1* mutants were homozygous (Figure 1A, Supplementary Table S2). The *dcl4-2e* mutant lines had point mutations in the DCL4 helicase domain that greatly reduced the accumulation of the 21 nt species of ta-siRNAs [38]. The genotyping PCR sequencing results of *dcl4-2e* mutants also indicated that there was a point mutation in *DCL4* that converts glutamic acid at position 583 to lysine, and the *dcl4-2e* mutants used in this study were homozygous (Figure 1B). To avoid transcriptional silencing induced by T-DNA insertion mutations, the T2 generation of RSV CP with Flag-Myc4 epitope transgenic plants on *dcl2-1* mutants was examined via western blot analysis. As shown in Figure 1C, relatively high levels of RSV CP expression were measured in transgenic plants, indicating that RSV CP loci were not transcriptionally silenced by T-DNA insertion in *dcl2-1* mutants. Transcriptional silencing was not a problem in the *dcl4-2e* mutant lines, which were not T-DNA insertion mutants. The expression of Flag-Myc4-CP was detected only in transgenic plants with the *dcl4-2e* background with an anti-Myc antibody (Figure 1D).

### 3.2. Plant DCL2 Is Indispensable for CP-Mediated RSV Resistance in Arabidopsis

**Figure 2:**
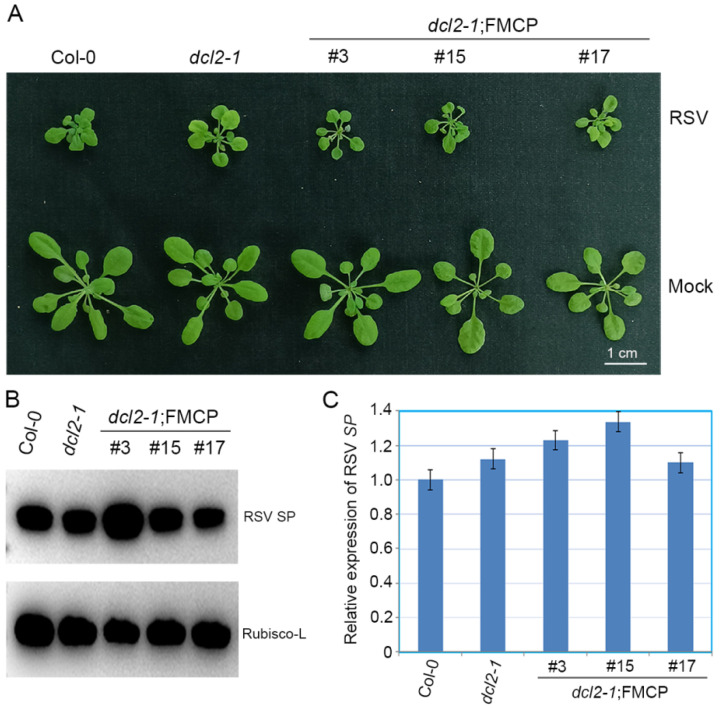
Evaluation of rice strip virus (RSV) resistance of RSV CP transgenic Arabidopsis plants with the *dcl2* mutant background. (**A**) Disease symptoms of mock-inoculated and RSV-infected CP transgenic plants with the *dcl2-1* mutant background (*dcl2-1*;FMCP-3, *dcl2-1*;FMCP-15, *dcl2-1*;FMCP-17) and *dcl2-1* and Col-0 Arabidopsis plants. Photographs were taken at 4 weeks post-inoculation. (**B**) Western blot analysis of RSV-encoded SP protein accumulation in RSV-infected CP transgenic plants with the *dcl2-1* mutant background (*dcl2-1*;FMCP-3, *dcl2-1*;FMCP-15, *dcl2-1*;FMCP-17) and *dcl2-1* and Col-0 Arabidopsis plants using an SP-specific antibody. The Rubisco-L protein level served as a loading control. Arabidopsis plants were all collected at 28 dpi. (**C**) qRT-PCR analysis of RSV SP mRNA transcription levels in RSV-infected CP transgenic plants with the *dcl2-1* mutant background (*dcl2-1*;FMCP-3, *dcl2-1*;FMCP-15, *dcl2-1*;FMCP-17) and *dcl2-1* and Col-0 Arabidopsis plants. Signal intensities for each transcript were normalized to those for *EF1-α*. Values are means ± SD (*n* = 3). The experiment was repeated twice with similar results.

To elucidate the function of DCL2 in RSV CP-mediated virus resistance, RSV CP transgenic Arabidopsis plants with the *dcl2-1* background were challenged with RSV infection by means of inoculation via viruliferous small brown planthoppers. Wild-type Col-0 and *dcl2-1* mutant plants were used as controls. In two independent experiments, RSV CP transgenic plants with the *dcl2-1* background (*dcl2-1*;FMCP) showed severe stunting disease symptoms similar to those of non-transgenic *dcl2-1* and Col-0 plants (Figure 2A). We assessed the extent of RSV SP mRNA transcripts using quantitative reverse-transcription PCR (qRT-PCR) and the abundance of RSV SP protein using western blot analysis. RSV SP mRNA and protein both accumulated at comparable levels in the *dcl2-1*;FMCP transgenic plants versus non-transgenic *dcl2-1* mutant and Col-0 plants (Figure 2B,C). Thus, DCL2 plays a primary role in the RSV CP-mediated virus resistance pathway. Considering that DCL2 is conserved in crop plants (Supplementary Figure S1), DCL2 may play an important role in crop plants-viruses interactions. 

### 3.3. RSV CP Transgenic Arabidopsis Plants with the dcl4-2e Background Show Immunity to RSV Infection

**Figure 3:**
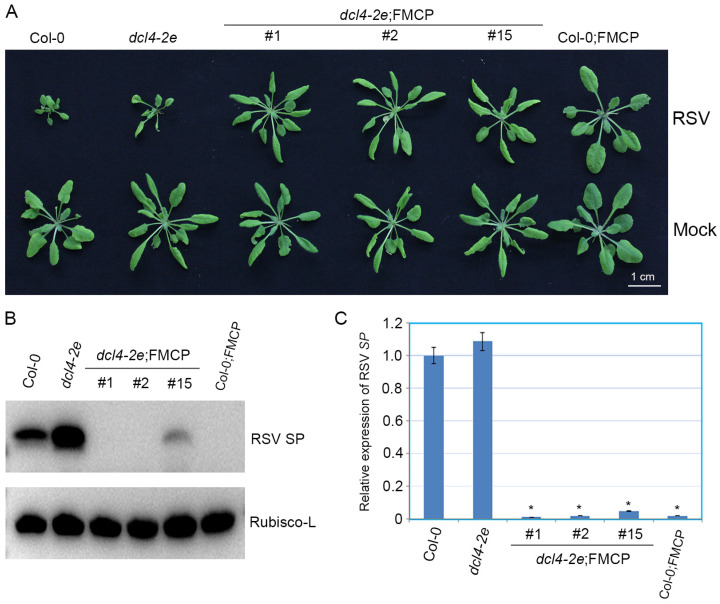
Examination of rice strip virus (RSV) resistance of RSV CP transgenic Arabidopsis plants with the *dcl4* mutant background. (**A**) Disease symptoms of mock-inoculated and RSV-infected CP transgenic plants with the *dcl4-2e* mutant background (*dcl4-2e*;FMCP-1, *dcl4-2e*;FMCP-2, *dcl4-2e*;FMCP-15) or wild-type background (Col;FMCP) and non-transgenic *dcl4-2e* and Col-0 Arabidopsis plants. Photographs were taken at 4 weeks post-inoculation. (**B**) Western blot analysis of RSV-encoded SP protein accumulation in RSV-infected CP transgenic plants with the *dcl4-2e* mutant background (*dcl4-2e*;FMCP-1, *dcl4-2e*;FMCP-2, *dcl4-2e*;FMCP-15) or wild-type background (Col;FMCP) and non-transgenic *dcl4-2e* and Col-0 Arabidopsis plants using a SP-specific antibody. The Rubisco-L protein level served as a loading control. Arabidopsis plants were all collected at 28 dpi. (**C**) qRT-PCR analysis for RSV *SP* mRNA transcription levels in RSV-infected CP transgenic plants with the *dcl4-2e* mutant background (*dcl4-2e*;FMCP-1, *dcl4-2e*;FMCP-2, *dcl4-2e*;FMCP-15) or wild-type background (Col;FMCP) and non-transgenic *dcl4-2e* and Col-0 Arabidopsis plants. Signal intensities for each transcript were normalized to those for *EF1-α*. Values are means ± SDs (*n* = 3). * *p* ≤ 0.05 (Student’s *t*-test). The experiment was repeated twice with similar results.

To further explore the roles of DCL4 in the RSV CP-mediated virus immunity pathway, we tested the antiviral responses of the RSV CP transgenic Arabidopsis plants with the *dcl4-2e* background (*dcl4-2e*;FMCP) by inoculating seedlings with RSV, using *dcl4-2e*, wild-type Col-0 as the virus susceptible control and CP transgenic plants with the Col-0 background (Col-0;FMCP) as the virus resistance control. In two independent trials, no RSV symptoms were observed in the *dcl4-2e*;FMCP and Col-0;FMCP transgenic Arabidopsis plants in contrast with the severe stunting symptoms in *dcl4-2e* and Col-0 plants (Figure 3A). Consistent with these observations, the accumulation of viral SP protein was nearly eliminated in the *dcl4-2e*;FMCP transgenic plants by means of western blot (Figure 3B), and the abundance of *SP* mRNA decreased in *dcl4-2e*;FMCP transgenic plants compared with that in *dcl4-2e* and Col-0 plants (Figure 3C). These results suggest that RSV CP induces viral resistance in the plants with the *dcl4-2e* background and that impairing DCL4 activity does not affect RSV CP-mediated viral resistance.

### 3.4. Deep Sequencing of Small RNA from RSV CP Transgenic Plants with dcl2-1, dcl4-2e and Wild-Type Backgrounds

**Figure 4:**
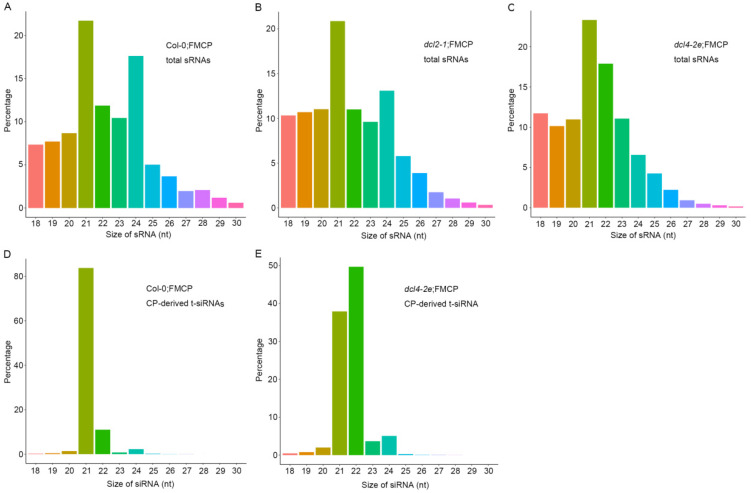
Size distribution of 18–30-nucleotide small RNAs. (**A**) Size distribution of total small RNA from RSV CP transgenic Arabidopsis plants with the wild-type background (Col;FMCP). (**B**) Size distribution of total small RNA from RSV CP transgenic Arabidopsis plants with the *dcl2-1* mutant background (*dcl2-1*;FMCP). (**C**) Size distribution of total small RNA from RSV CP transgenic Arabidopsis plants with the *dcl4-2e* mutant background (*dcl4-2e*;FMCP). (**D**) Size distribution of CP-derived t-siRNAs from RSV CP transgenic Arabidopsis plants with the wild-type background (Col;FMCP). (**E**) Size distribution of CP-derived t-siRNAs from RSV CP transgenic Arabidopsis plants with the *dcl4-2e* mutant background (*dcl4-2e*;FMCP).

We previously showed that RSV CP-mediated virus resistance was associated with transgene-derived 21–24 nt small RNAs on CP loci (t-siRNAs) [35]. To further explore the function of DCL2 and DLC4 in regulating CP-derived t-siRNAs, we conducted small RNA deep sequencing with total RNA prepared from *dcl2-1*;FMCP and *dcl4-2e*;FMCP transgenic plants using Col-0;FMCP as a control. Each sample generated 11–14 million clean reads ranging from 18 to 30 nt (Table 1). Within the *dcl2-1*;FMCP and Col-0;FMCP samples, the size distributions of total small RNAs were similar; the dominant size was 21 nt, which was followed by 24 nt and 22 nt (Figure 4A,B). However, in *dcl4-2e*;FMCP, the most dominant sizes of total small RNAs were 21 nt, followed by 22 nt, and the number of 24 nt small RNAs was reduced, while the number of 22 nt small RNAs was increased compared to those in *dcl2-1*;FMCP and Col-0;FMCP plants (Figure 4C). These results indicate that when DCL4 function was impaired, endogenous 22 nt small RNAs were excessively produced by DCL2.

Furthermore, we mapped clean small RNA (18–30 nt) reads to the CP transgenic sequence and obtained CP-derived siRNAs. In Col-0;FMCP and *dcl4-2e*;FMCP transgenic plants, we identified 82,012 and 271,849 CP-derived t-siRNAs, accounting for 0.84% and 2.78% of the total, respectively, whereas only 221 reads matched the CP sequence in *dcl2-1*;FMCP plants (Table 1). The sequencing results indicate that the *dcl2* mutation eliminated the accumulation of CP-derived t-siRNAs from transgenic sequences, while in contrast, mutations in DCL4 promoted the production of CP-derived t-siRNAs to a certain extent. Size distribution analysis showed that CP-derived t-siRNAs were predominantly 21 and 22 nt, accounting for 83.76% and 11.00% of the total, respectively, in Col-0;FMCP plants (Figure 4D). However, in *dcl4-2e*;FMCP transgenic plants, the dominant sizes were 22 nt and 21 nt, accounting for 49.68% and 37.92% of the total, respectively (Figure 4E), suggesting that mutations in DCL4 promoted a dramatic shift in the production of CP-derived t-siRNAs from 21 to 22 nt.

**Figure 5:**
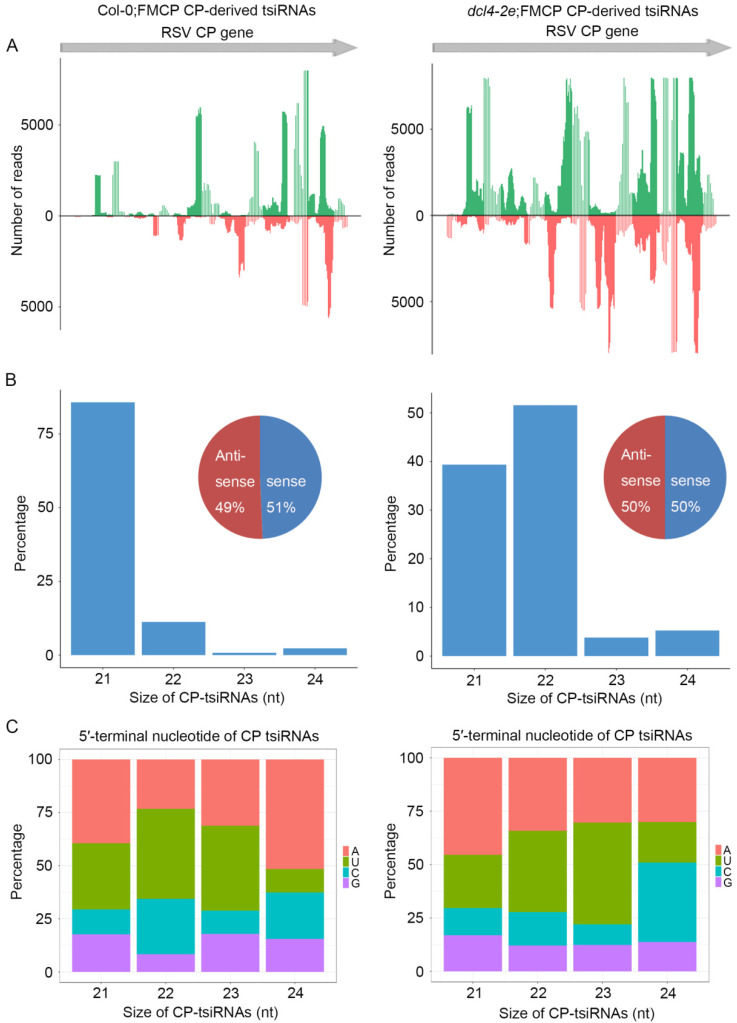
Characterization and comparison of 21–24 nt transgenic-derived small interfering RNA (t-siRNA) mapped to the CP sequence in RSV CP transgenic in wild-type background Arabidopsis plants (Col;FMCP) (left) and in *dcl4-2e* mutant background Arabidopsis plants (*dcl4-2e*;FMCP) (right). (**A**) Distribution of t-siRNAs along the CP sequence in both positive (blue) and negative (red) polarity. (**B**) Size distribution of t-siRNAs. Pie graph showing the percentage of sense and antisense t-siRNAs. (**C**) 5′-terminal nucleotide frequency of 21–24 nt t-siRNAs.

To further decipher the regulatory functions of t-siRNAs, we further compared the canonical 21–24 nt t-siRNAs from the CP transgenic sequence generated from Col-0;FMCP and *dcl4-2e*;FMCP transgenic plants. The 21–24 nt CP-derived t-siRNA reads were mapped to the CP sequence to explore their origin. As shown in Figure 5A, in Col-0;FMCP plants, the proportions of CP t-siRNAs from both polarities were almost continuous, but some genomic regions exhibited higher mapping frequencies. Similarly, *dcl4-2e*;FMCP plants also showed a very similar profile of CP t-siRNAs with peaks in the same position, although the t-siRNA peak read number was much higher than that of Col-0;FMCP (Figure 5A). The size distributions of CP t-siRNAs showed that 21 nt was the most dominant size, representing 80% of the total t-siRNAs in Col-0;FMCP plants (Figure 5B). However, in *dcl4-2e*;FMCP plants, 22 nt and 21 nt were the most dominant sizes, representing 50% and 37% of the total, respectively (Figure 5B). To investigate the origin of CP t-siRNAs, we analyzed the strand specificity and locations of CP t-siRNAs. As shown in Figure 5A,B, Col-0;FMCP and *dcl4-2e*;FMCP plants both showed similar CP t-siRNA distribution profiles, producing almost equivalent proportions of sense and antisense t-siRNAs. The 5′ terminal nucleotides were characterized for 21–24 nt CP t-siRNAs. The U/A were the most abundant 5′ nucleotides in all four sizes of both Col-0;FMCP and *dcl4-2e*;FMCP transgenic plants (Figure 5C). The characterization of CP t-siRNAs in CP transgenic Arabidopsis plants with the *dcl2-1* or *dcl4-2e* mutant backgrounds indicates that the accumulation of CP-derived t-siRNAs requires DCL2 but not DCL4, providing evidence that DCL2 plays an essential role in CP-mediated virus resistance.

## 4. Discussion

Plant viruses cause yield losses in agricultural crops worldwide, affecting the progress toward global food security. Therefore, there is an urgent need to seek environmentally friendly and effective virus control strategies that are easier to deploy to control viral diseases. Since the first discovery in the late 1980s that tobacco mosaic virus (TMV) coat protein (CP) confers resistance to the cognate virus in transgenic tobacco plants, a new avenue for virus disease control has been provided [40]. Virus CP-mediated resistance has been found to be highly effective for many different plants against several different viruses [41]. Subsequent studies have shown that the mechanisms underlying CP-mediated virus resistance could differ from one plant virus system to another. However, it is believed that CP-mediated virus resistance is often due to an RNA silencing resistance mechanism [42]. CP transgene integration events result in sufficient quantities of double-strand RNAs (dsRNAs), which are processed by a DCL enzyme into small RNAs. These small RNAs program AGO proteins in RISCs to target and degrade viral genomic RNA. However, due to the presence of multiple copies of the DCL and AGO proteins in plants, the detailed molecular mechanisms underlying CP-mediated virus resistance are not fully understood. In this study, we showed that Arabidopsis DCL2, rather than DCL4, plays a primary role in RSV CP-mediated resistance. First, we generated RSV CP transgenic Arabidopsis with a *dcl2* or *dcl4* mutant background. Second, a viral infection assay indicated that RSV CP transgenic plants in *dcl2* mutants (*dcl2-1*;FMCP) exhibited similar sensitivity to virus infection when compared to non-transgenic plants; nevertheless, CP overexpression in *dcl4* mutants (*dcl4-2e*;FMCP) provided immunity to viral infection. Third, by using deep sequencing analysis, accumulation of t-siRNA from CP loci was eliminated in *dcl2-1*;FMCP transgenic plants, and *dcl4-2e*;FMCP plants promoted the production of 22 nt t-siRNAs from CP loci. Our results strongly indicate that DCL2 but not DCL4 is required for RSV CP-mediated resistance and the accumulation of CP-derived small RNAs in Arabidopsis.

In Arabidopsis plants, there are four DCL genes that cleave stem-loop or double-stranded (ds) RNA precursors into 21–24 nt small RNAs [1]. DCL1 primarily processes hairpin RNA into a 21 nt miRNA that is typically involved in regulating developmental genes at the post-transcriptional level [43]. The main activity of DCL3 is processing long dsRNA into 24 nt siRNAs that guide the RNA-directed DNA methylation (RdDM) pathway [44]. DCL4 and DCL2 produce 21 nt and 22 nt siRNAs from RDR6-dependent dsRNA involved in transgene PTGS and resistance against the virus pathway. Importantly, DCL2 generates 22 nt siRNAs, especially from NIA1/2, which is involved in plant adaptation to environmental stress [45]. Furthermore, our genetic analysis described here uncovers the crucial role of DCL2 in the virus CP-mediated resistance pathway.

In general, it is widely accepted that virus CP-mediated resistance is similar to sense transgene-induced silencing with regard to its mechanism [42]. Although there is a significant difference between these two types of RNA silencing, such as the high level of CP protein accumulation detected in our CP-mediated resistance system (Figure 1C,D), the accumulation of transgene mRNA or protein is very low in the sense transgene-induced silencing system [46]. RNA silencing pathways have shown that DCL2, DCL3 and DCL4 can compete for the same dsRNA substrates [4,47]. Our results show that in wild-type plants, the majority of CP-derived t-siRNAs were 21 nt in size (Figure 4), suggesting that DCL4 appears to be the most active enzyme on the dsRNA substrates produced by CP transgenes. This finding is further illustrated by the fact that in *dcl4* mutant plants, the 22 nt CP-derived t-siRNAs were significantly increased (Figure 4). Our results are in line with the previous findings that in the sense transgene-induced silencing system, the action of DCL2 is counteracted when a functional DCL4 is present [11,48]. Transgenic studies have also demonstrated that DCL2 plays an essential role in sense transgene-induced silencing [11,48]. We reached similar conclusions using the RSV CP-mediated virus system. Indeed, CP transgenic plants in the *dcl2* mutant background showed similar virus susceptibility to non-transgenic plants and the concomitant absence of CP-derived t-siRNAs of all sizes (Figure 2, Table 1). This suggests that DCL2 or the 22 nt class of t-siRNAs it produces is necessary for the participation of DCL4 and other DCL enzymes in the generation of t-siRNAs on dsRNA from the CP transgene. In fact, 22 nt siRNAs rather than 21 nt siRNAs trigger secondary siRNA production by recruiting a dsRNA binding protein, SGS3, and an RNA-dependent RNA polymerase (RDR) protein [1]. These results indicate that the amount of 21 nt primary t-siRNAs generated by DCL4 is insufficient to achieve virus resistance in CP transgenic plants and that sufficient 21 nt secondary t-siRNAs triggered by 22 nt t-siRNAs are required to achieve the complete degradation of target virus genomic RNAs. In conclusion, the present work and previous studies all demonstrate that DCL2 plays an essential, as opposed to redundant, role in RSV CP-mediated resistance and sense transgene-induced silencing in Arabidopsis [11,48]. 

Based on small RNA-Seq, our present work also showed that the amount of CP-derived 21 nt t-siRNAs was not significantly different in the *dcl4-2e* plant compared with the wild type (Figure 4D,E, Table 1), which is consistent with an earlier report for sense transgene-mediated silencing [49]. This result indicates that with the inactivation of DCL4, DCL1 activity-dependent 21 nt t-siRNA biogenesis was enhanced. In antiviral immunity, all four DCLs are involved in the production of virus-derived siRNAs (vsiRNAs). The accumulation of 21 nt viRNAs was detected in the *dcl2*/*dcl3*/*dcl4* mutant plants infected with viruses, indicating the role of DCL1 in viRNA generation. Specifically, DCL1 produces 21 nt v-siRNAs from the leader region of cauliflower mosaic virus (CaMV), suggesting that DCL1 may be involved in small RNA-mediated defense [4]. Further studies are needed to elucidate the role of Arabidopsis DCL1 in CP-mediated virus resistance.

In Arabidopsis, AGO1 and AGO2 have been shown to play roles in antivirus defense [7] and bind small RNAs that display uridine and adenosine at their 5′ ends, respectively [50]. Additionally, hypomorphic *ago1* mutants, deficient in the sense transgene, mediate silencing and are hypersusceptible to infection by cucumber mosaic virus (CMV) [51], indicating that AGO1 plays crucial roles in sensing transgene-mediated silencing and virus defense. We also observed the preferential occurrence of uridine or adenosine residues at the 5′ terminus in CP-derived t-siRNAs (Figure 5C), showing the conserved AGO complexes binding t-siRNA for RNA silencing and virus defense. Clearly, the function of AGO1 and AGO2 in RSV CP-mediated virus resistance needs to be further investigated.

Finally, our genetic and small RNA-seq analysis described here demonstrates that DCL2 plays a crucial role in RSV CP-mediated virus resistance. We anticipate that the identification and characterization of DCL2′s function in RSV CP-mediated virus resistance developed here will help in the development of a deep understanding of RNA-based antiviral immunity in RSV-Arabidopsis interactions.

## Figures and Tables

**Table 1:** Three libraries constructed with small RNAs from RSV CP transgenic Arabidopsis plants with the wild-type background (Col;FMCP), with the *dcl2-1* mutant background (*dcl2-1*;FMCP) and with the *dcl4-2e* mutant background (*dcl4-2e*;FMCP).

Library	Clean Reads	Total 18–30 nt Reads	Number of Reads on CP	Percentage of CP Reads (%)
Col-0;FMCP	12,742,300	9,770,500	82,012	0.84
*dcl2-1*;FMCP	14,707,016	9,295,105	221	0.00
*dcl4-2e*;FMCP	11,574,998	9,770,500	271,849	2.78

## Data Availability

No new data were created or analyzed in this study. Data sharing is not applicable to this article.

## Supplementary Materials

The following supporting information can be downloaded at https://www.mdpi.com/article/10.3390/v15112239/s1, Figure S1. Dendrogram image of DCL2 proteins from Arabidopsis and some agriculturally important crops. A dendrogram image constructed from DCL2 full-length amino acid sequences from the Arabidopsis and some agriculturally important crops. The Arabidopsis DCL4 full-length sequences were included as an outgroup. The dendrogram image was constructed by TBtools software using the Neighbor-Joining (NJ) method with 5000 bootstrap numbers. All the protein sequences were downloaded from NCBI (National Center for Biotechnology Information). Table S1. Primer sequences used for genotyping and testing the viral infection. Table S2. PCR products sequences for *dcl2-1* mutants genotyping.
