# Supplementary material for: DICER-LIKE2 Plays a Crucial Role in Rice Stripe Virus Coat Protein-Mediated Virus Resistance in Arabidopsis
Source: Viruses. 2023 Nov 10;15(11):2239. doi: 10.3390/v15112239 (PMC10675384; doi:10.3390/v15112239)
Supplement: Supplementary file 1 [file viruses-15-02239-s001.zip › Table S1.pdf]

Table S1. Primers sequences used for genotyping and testing the viral infection.

Gene	Primers	Product Size (bp)	tests
DCL2	5’- TGAATCATCTGGAAGAGGTGG -3’<br>5’- CTTCACAGGAGTTTTTGGCTG -3’	1060	genotyping
DCL4	5’- TGAAGAGCATGTCAAGAAGGAG -3’<br>5’- GAGCACGACCTCTGGACTGT -3’	351	genotyping
LB1.3	5’- ATTTTGCCGATTTCGGAAC -3’	-	genotyping
SP	5’-TTGTCACTCATTCTTATCACACCTG-3’<br>5’-TTCTTCCACACTTTCTCATACTCTT-3’	237	qRT-PCR
EF1-a	5’-GGCTGCTGAGATGAACAA-3’<br>5’-GTGGTGGAGTCAATGATAAG-3’	225	qRT-PCR
