# Supplementary material for: DICER-LIKE2 Plays a Crucial Role in Rice Stripe Virus Coat Protein-Mediated Virus Resistance in Arabidopsis
Source: Viruses. 2023 Nov 10;15(11):2239. doi: 10.3390/v15112239 (PMC10675384; doi:10.3390/v15112239)
Supplement: Supplementary file 1 [file viruses-15-02239-s001.zip › Table S2.pdf]

Table S2. PCR products sequences for *dcl2-1* mutants genotyping.
Arabidopsis genome sequences were in black font and T-DNA vector sequences were in red font.

dcl2-LP/ *dcl2*-RP PCR product sequence
TTGTTTGCTAACATTTAGTGATTGAAGTTTCATTCATTTCTTATATTTCCTCACCTCTTTTGTTCTTTTCATGTGT<br>AAATAGGTCTTAATTGGAGTGTTGCAAACATAAATGGAAATGCGGAGGCAGGTCTCCTAACTTTAAAAACTG<br>TCTGCCTCATTGAGACTCTTCTTGGTTATAGGTTTGGATTGCATGCACACATTTTTGTTTATGCTTATTATATTG<br>GAGTTAGATATGACGATCTGAAATGATAAACCTTGAATGTTGGACATTACTTATATCAATACATATTGGAGTAC<br>AATGGTTATTTAAAATCTGTGTTGTTTTCCAGTTCTATTTCTCTGAATTTTTATTGATACAGCTCCTTGGAGAAC<br>ATACGGTGCATCATTTTTGTGGATAGGGTGATAACAGCCATCGTTCTGGAATCCCTTTTGGCTGAGATTCTTC<br>CAAACTGTAATAACTGGAAAACCAAGTACGTTGCAGGAAATAACTCTGGTCTGCAAAATCAAACTCGGAAG<br>AAGCAAAATGAAATTGTGGAAGACTTCCGGAGAGGCTTGGTATGGTCAAATTACTGTGAACTGTTGTTTTAT<br>TTGCTTACTGTTATCTTTATTTCTGAGTAACCTGTTCGTATTCGTAGGTTAACATCATTGTAGCAACATCTATTCT<br>AGAGGAGGGTCTAGATGTTCAAAGTTGCAACCTGGTTATCAGATTTGACCCTGCATCCAACATTTGCAGTTT<br>CATACAGTCTCGTGGGCGTGCTAGAATGCAAAATTCAGATTATTTGATGATGGTGGAAAGGTCTGTTACATAT<br>TTGGCTGGTCTTCAGAGAGTTTACGATTTGTTTTGAAAACAATAGCTCCATGCTTAAAAAAAATATAATTCTTT<br>CT
LB1.3/ *dcl2*-LP PCR product sequence
GTGGATCGGATTTCGCCTGCTGGGGCAACCAGCGTGGACCGCTTGCTGCAACTCTCTCAGGGCCAGGCGG<br>TGAAGGGCAATCAGCTGTTGCCCGTCTCACTGGTGAAAAGAAAAACCACCCCAGTACATTAAAAACGTCCG<br>CAATGTGTTATTAAGTTGTCTAAGCGTCAATTTGTTTACACCACAATATATCCTGGAGAACATACGGTGCATCA<br>TTTTTGTGGATAGGGTGATAACAGCCATCGTTCTGGAATCCCTTTTGGCTGAGATTCTTCCAAACTGTAATAA<br>CTGGAAAACCAAGTACGTTGCAGGAAATAACTCTGGTCTGCAAAATCAAACTCGGAAGAAGCAAAATGAAA<br>TTGTGGAAGACTTCCGGAGAGGCTTGGTATGGTCAAATTACTGTGAACTGTTGTTTTATTTGCTTACTGTTAT<br>CTTTATTTCTGAGTAACCTGTTCGTATTCGTAGGTTAACATCATTGTAGCAACATCTATTCTAGAGGAGGGTCT<br>AGATGTTCAAAGTTGCAACCTGGTTATCAGATTTGACCCTGCATCCAACATTTGCAGTTTCATACAGTCTCGT<br>GGGCGTGCTAGAATGCAAAATTCAGATTATTTGATGATGGTGGAAAGGTCTGTTACATATTTGGCTGGTCTTC<br>AGAGAGTTTACGATTTGTTTTGAAAACAATAGCTCCATGCTTAAAAAAAATATAATTCTTTCTCACTCATTGCC<br>ACTGGCAGCGGAGATCTGTTAACACAATCTCGATTAATGAAATATCTTTCTGGTGGGAAAAGAATGCGCGAA<br>GAGTCTTTGGATCATTCTCTTGTTCCCTGTCCACCTCTTCCAGATGATTCA
LB1.3/ *dcl2*-RP PCR product sequence
CTTCACAGGAGTTTTTGGCTGAGATACCTCAAGGTGGTTTTTTGTTTGCTAACATTTAGTGATTGAAGTTTCA<br>TTCATTTCTTATATTTCCTCACCTCTTTTGTTCTTTTCATGTGTAAATAGGTCTTAATTGGAGTGTTGCAAACATA<br>AATGGAAATGCGGAGGCAGGTCTCCTAACTTTAAAAACTGTCTGCCTCATTGAGACTCTTCTTGGTTATAGGT<br>TTGGATTGCATGCACACATTTTTGTTTATGCTTATTATATTGGAGTTAGATATGACGATCTGAAATGATAAACCT<br>TGAATGTTGGACATTACTTATATCAATACATATTGGAGTACAATGGTTATTTAAAATCTGTGTTGTTTTCCAGTT<br>CTATTTCTCTGAATTTTTATTGTGGTGTAAACAAATTAAGGGAGAATTAAGGGATATATTGTGGCTAAGCAAAT<br>ACTATTAACGTAAAACTATCAGTATATATTGCGCCTTCAGGATATATTGTGGTGTAAACAAATTGACGCTTAGA<br>CAACTTAATAACACATTGCGGACGTTTTTAATGTACTGGGGTGGTTTTTCTTTTCACCAGTGAGACGGGCAA<br>CAGCTGATTGCCCTTCACCGCCTGGCCCTGAGAGAGTTGCAGCAAGCGGTCCACGCTGGTTTGCCCCAGCA<br>GGCGAAAATCCTGTTTGATGGTGGTTCCGAATCCGGCAAAAT
