# Supplementary figures and images for: DICER-LIKE2 Plays a Crucial Role in Rice Stripe Virus Coat Protein-Mediated Virus Resistance in Arabidopsis
Source: Viruses. 2023 Nov 10;15(11):2239. doi: 10.3390/v15112239 (PMC10675384; doi:10.3390/v15112239)

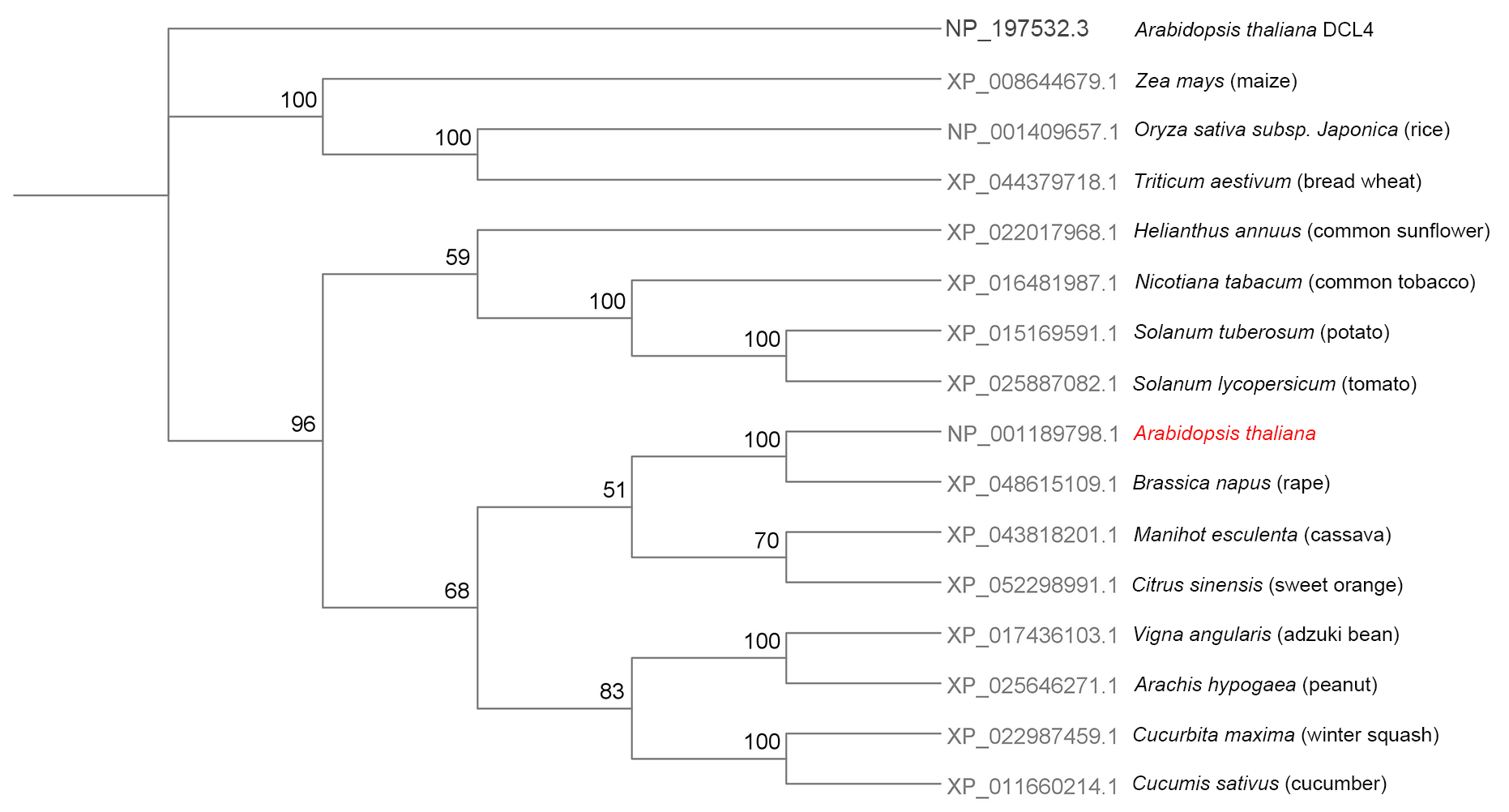

Supplement: Supplementary file 1 [file viruses-15-02239-s001.zip › Fig S1.tif]
